# Role of advanced imaging in primary hepatic neuroendocrine tumor with borderline raised AFP and negative chromogranin staining: A case report

**DOI:** 10.1016/j.ijscr.2024.110647

**Published:** 2024-11-23

**Authors:** Asma Fatima, Suresh Chandra, Saubia Fatima, Mohammed Yasir Izhar, Syed Faqeer Hussain Bokhari, Asma Iqbal

**Affiliations:** a Department of General Surgery, Vikarabad Government Medical College, India; b Department of Surgical Gastroenterology, Deccan College of Medical Sciences, India; c Department of Radiology, Deccan College Of Medical Sciences, India; d MBBS, Deccan College of Medical Sciences, India; e MBBS, King Edward Medical University, Mayo Hospital, Lahore, Pakistan

**Keywords:** Primary hepatic neuroendocrine tumor (PHNET), Advanced imaging, Immunohistochemistry (IHC), Surgical resection, Borderline elevated alpha-fetoprotein, Positron emission tomography-computed tomography

## Abstract

**Background:**

Primary hepatic neuroendocrine tumors (PHNETs) are rare, accounting for approximately 0.3 % of all neuroendocrine tumors (NETs) and are often difficult to diagnose due to their nonspecific symptoms and imaging features. Standard diagnostic and treatment protocols are lacking due to their rarity, but imaging and immunohistochemistry (IHC) remain key tools for diagnosis.

**Case report:**

A 68-year-old male presented with abdominal discomfort and loss of appetite. Imaging revealed a large exophytic mass in the left lobe of the liver. After ruling out extrahepatic primary sources, a left lobe hepatectomy was performed. Histopathology confirmed the diagnosis of PHNET, with positive IHC staining for synaptophysin and CK-7. Postoperative PET-CT ruled out any distant metastases. The patient had an uneventful recovery.

**Discussion:**

PHNETs are believed to originate from ectopic neuroendocrine cells in the liver, though several theories exist. Imaging alone cannot conclusively diagnose PHNETs, as they mimic other hepatic tumors like hepatocellular carcinoma. Histopathological examination, along with IHC markers like chromogranin and synaptophysin, is essential for diagnosis. Surgical resection remains the treatment of choice, with good outcomes despite the risk of recurrence. Non-surgical therapies, such as chemotherapy or ablation, are under investigation but lack consensus.

**Conclusion:**

PHNETs are rare and challenging to diagnose, requiring imaging and IHC for confirmation. Surgery offers the best prognosis, making personalized, surgery-centered treatment plans essential for management. Comprehensive follow-up, including functional imaging, is necessary to monitor recurrence or metastasis.

## Highlights

• This case highlights a rare Primary Hepatic Neuroendocrine Tumor (PHNET), accounting for only 0.3% of all NETs
• Imaging revealed a large exophytic liver mass; after ruling out extrahepatic sources, a left lobe hepatectomy was performed, leading to an uneventful recovery.
• Histopathology confirmed PHNET with positive synaptophysin and CK-7 staining, but negative chromogranin, aiding in precise diagnosis.
• PET-CT and Ga-68 DOTANOC PET/CT scans ruled out metastases and identified a possible postoperative seroma, emphasizing the importance of imaging in monitoring.
• Surgical resection remains the best option for PHNET, offering both diagnostic confirmation and curative potential, despite the risk of recurrence

## 1 Introduction

Neuroendocrine tumors (NETs) are a group of rare and highly heterogeneous malignant disorders derived from the diffuse neuroendocrine system [1]. NETs account for 1–2 % of gastrointestinal malignancies. Although the liver is the most frequent location for the spread of these tumors, primary hepatic neuroendocrine tumors (PHNETs) are extremely uncommon [2]. Edmonson reported the first case of PHNET in 1958 [3]. Since then, almost 150 cases have been documented in the literature, making up around 0.3 % of all neuroendocrine tumors [4]. Owing to their rarity, there is a lack of standard diagnostic criteria and therapeutic regimens for PHNETs. Certain studies indicate that imaging modalities and histopathological confirmation are necessary to diagnose PHNETs [5,6]. Immunohistochemical staining studies reveal chromogranin A (CgA) and synaptophysin (Syn) as two unique markers for NETs [7,8]. To rule out extrahepatic NETs, imaging techniques such as computed tomography (CT), Magnetic Resonance Imaging (MRI), and positron emission tomography-CT (PET-CT) are helpful [5,6]. The only treatment option available with considerable expectation of cure is surgical resection of the tumor [9,10].

## 2 Case presentation

A 68-year-old man presented to the emergency department with complaint of abdominal fullness with discomfort since 4 days and loss of appetite for 2 weeks. He was a known case of hypertension and was stabilized on amlodipine (2.5 mg) for one year. General physical examination and baseline investigations were inconclusive. Minimal right upper quadrant tenderness was observed on palpitation. There was no rebound tenderness or guarding. Laboratory tests were significant for GGT levels of 264 U/l (normal 11–49 U/l). Liver function tests showed elevated serum ALP, SGOT and SGPT levels. Tumor marker tests showed borderline elevated AFP levels of 17.5 ng/ml (normal 0–15 ng/ml).

**Fig. 1:**
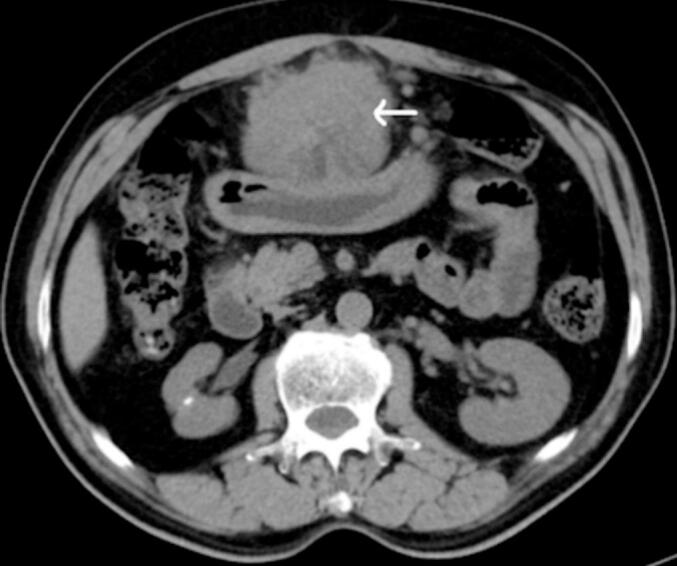
CECT abdomen, axial image showing an irregular exophytic mass (arrow) with both solid and cystic components noted in left lobe of liver (segment III) measuring 76 × 64 × 93 mm.

**Fig. 2:**
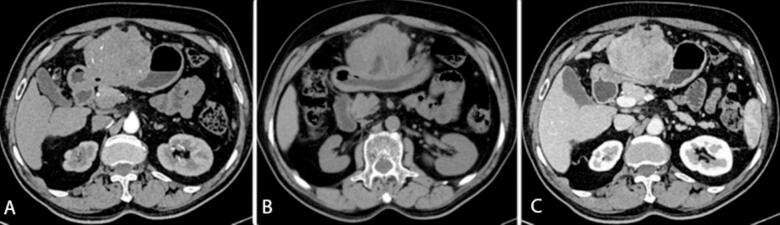
Triphasic CECT abdomen, axial image showing minimal heterogeneous enhancement during arterial (A) phase which was increasing during the portal (B) and venous (C) phases.

Ultrasound scan of abdomen and pelvis demonstrated mildly altered echotexture of liver and non-obstructive right renal calculus. Triple phase Contrast-Enhanced Computed Tomography (CECT) scan of abdomen revealed an irregular exophytic lesion showing both solid and cystic components in left lobe of liver (segment III) measuring 76 × 64 × 93 mm (Fig. 1). Minimal heterogeneous enhancement during arterial phase was observed which was increasing during the portal and venous phases (Fig. 2). The lesion was extending till the umbilicus causing mass effect over the stomach and displacing it posteriorly.

Neoplastic etiology was suspected and biopsy was not performed to avoid tumor seeding. An upper and lower gastrointestinal endoscopy, a Technetium-99 m bone scan, and a negative chest computed tomography scan were part of further testing to ascertain primary tumor or other metastatic locations. The decision to surgically resect the lesion was opted and uneventful left lobe hepatectomy was performed. Drain was removed on day 7. Rest of the post-operative period was uneventful. AFP levels remained same post-operatively indicating no association of the tumor with borderline raised AFP levels.

**Fig. 3:**
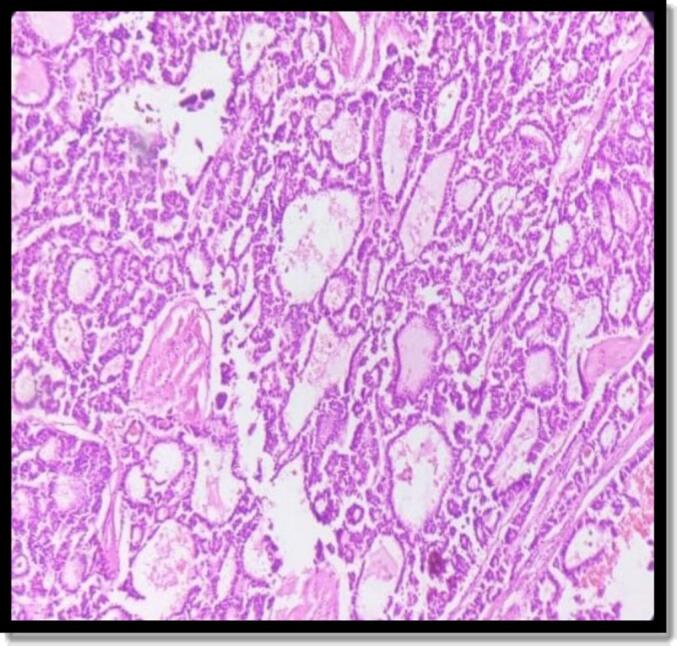
HPE of specimen showing trabeculae of tumor cells and pseudoacinar pattern.

**Fig. 4:**
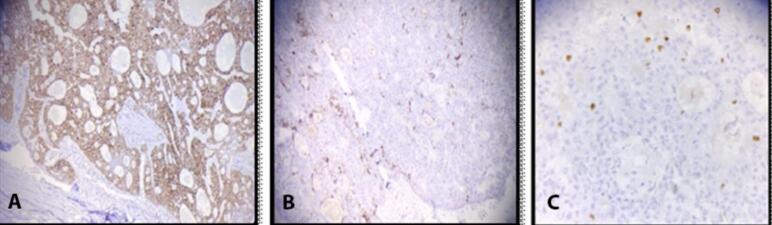
IHC staining positive for Syn (A), CK-7 (B) and Ki-67 (C).

Histopathological Examination (HPE) of the specimen measuring 13 × 8 × 6 cm revealed trabeculae of tumor cells, 5–6 layers thick with vesicular nuclei and pseudoacinar pattern. Focally, there was nuclear atypia with intervening areas showing fibrocollagenous tissue. Hemorrhagic areas and vascular congestion were noticed (Fig. 3). Immunohistochemical (IHC) staining was positive for Syn, CK-7 and Ki-67 (Fig. 4). Chromogranin, NSE and TTF1 staining was negative. Based on these findings, the tumor was labelled as hepatic neuroendocrine tumor (grade II).

A Positron Emission Tomography-Computed Tomography (PET-CT) scan was conducted eight weeks postoperatively to evaluate for any potential distant metastases or identify a primary tumor site. The results indicated no evidence of metabolically active lesions elsewhere in the body, ruling out distant disease.

**Fig. 5:**
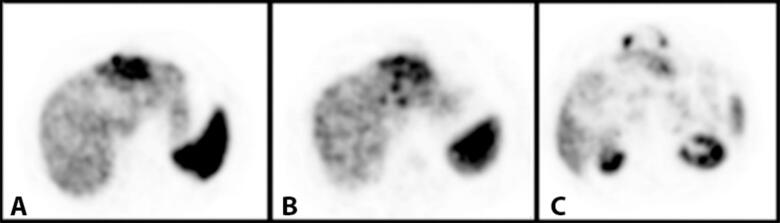
Liver shows increased patchy Ga-68 DOTATE uptake in the regions of heterogeneity. Spleen shows normal physiological Ga-68 DOTATE uptake. No focal lesions were noted.

Subsequently, a 68Ga-DOTANOC PET/CT scan was performed for further assessment. This scan revealed a well-defined, thick-walled cystic collection localized within the surgical bed. The collection exhibited mild peripheral tracer uptake, suggestive of a residual or reactive process. Anatomically, the lesion was found to abut the stomach inferiorly, causing a potential mass effect. Furthermore, an ill-defined communication was identified between the cystic collection and the adjacent biliary radicle. This finding raised the differential diagnosis of a seroma, which is a common postoperative fluid collection. However, the possibility of a biloma—a bile leak-induced collection—could not be conclusively excluded and warranted further clinical correlation and follow-up imaging (Fig. 5).

## 3 Discussion

NETs originate from neuro-ectodermal cells, which spread from the neural crest throughout the body during embryogenesis. NETs frequently metastasize to the liver, with some studies reporting that >80 % of all NET patients had hepatic metastases at the time of diagnosis [11]. However, PHNETs are rare because neuro-ectodermal cells do not frequently move to the liver [12]. There are several possibilities to explain the pathophysiology of PHNETs because these progenitor cells are not naturally occurring in the liver. According to Hsueh et al., the genesis of PHNET formation might be ectopic adrenal or pancreatic tissue [13]. According to Alpert et al., argentaffin cells seen in the bile duct epithelium may be the cause of malignant malignancies [14]. In both of these instances, intestinal metaplasia, which in turn predisposes to the formation of NETs, may be brought on by persistent biliary system inflammation [15]. PHNETS are hard to distinguish on imaging from other tumors with a healthy are rare blood supply, such as HCC, hepatic adenoma, and FNH. Comprehensive imaging examinations before surgery and ongoing imaging examinations during postoperative follow-ups are crucial for diagnosing extrahepatic neuroendocrine tumors. PHNETs primarily manifest between the ages of 40 and 60, and no gender bias has been noted [9]. Just 6.8 % of PHNET patients exhibit the characteristic carcinoid syndrome while the majority of patients do not exhibit any particular symptoms [4]. As a result, early diagnosis of PHNET based only on symptoms is challenging. Moreover, unlike hepatocellular carcinomas, PHNETs are not linked to cirrhosis or hepatitis, and the existence of PHNETs results in hepatic tumor markers like AFP and CEA within the normal range [16].

Currently, there are no specific imaging features, and cases are diagnosed retrospectively. In addition to non-contrasted Computed Tomography (CT) scans showing low-density masses, some of which have a cystic component, the diagnosis is based on laboratory findings showing negative serum alpha-fetoprotein levels and negative results for other traditional tumor markers (carcinoembryonic antigen, CA125, and CA19–9). Dynamic contrast CT indicates early-phase increased masses and late-phase low-density masses. On magnetic resonance imaging (MRI), PHNEC typically manifests as a large dominant hypervascular mass accompanied by satellite nodules, low intensity on T1-weighted images and high intensity on T2-weighted images, rapid washout and capsular enhancement on dynamic MRI, and restricted diffusion on diffusion-weighted images [17]. The diagnosis may be confirmed by immunohistochemistry where the cells present a strong positivity for neurosecretory markers such as chromogranin, synaptophysin, neuron-specific enolase, and S-100 protein [18].

Given the rarity of the finding, a thorough investigation for an extrahepatic primary carcinoid tumor is necessary. There is currently no agreement on how to manage PHNETs. Some studies claim that the only treatment that could provide a definitive cure is surgical excision. Despite recurrences, the resectability rate and overall survival are good [19,20].

## 4 Conclusion

Imaging modalities such whole-body PET CT scan and Ga-68 DOTANOC PET CT scan may be used to rule out extrahepatic neuroendocrine tumors and establish the diagnosis of PHNETs. AFP levels might be raised but utilization of AFP in PHNETs is poorly understood currently. HPE and IHC staining provide a definitive diagnosis. Surgery-centered, comprehensive, and personalized treatment plans should be developed for cases in which PHNETs have been verified. Surgical resection is the first line of treatment for individuals with locally treatable lesions since it not only improves prognosis but also yields a pathological diagnosis via HPE and IHC staining.

## Methods

The work has been reported in line with the SCARE criteria [21].

## CRediT authorship contribution statement

**Asma Fatima:** Data acquisition, Writing of article
**Suresh Chandra:** Study conception, Writing of article
**Saubia Fatima:** Data acquisition, Writing of article
**Mohammed Yasir Izhar:** Study conception
**Syed Faqeer Hussain Bokhari:** Writing of article
**Asma Iqbal:** Writing of article.

## Informed consent

Written informed consent was obtained from the patient's parents/legal guardian for publication and any accompanying images. A copy of the written consent is available for review by the Editor-in-Chief of this journal on request.

## Ethical approval

As it's a case report, it is exempted from ethical approval by the Institutional Board of Review, Deccan College of Medical Sciences, India.

## Guarantor

Asma Fatima.

## Research registration number

N/A.

## Funding

None.

## Declaration of competing interest

None to disclose.
